# Association of FAS gene polymorphisms with the risk of noise-induced hearing loss in Chinese occupational workers

**DOI:** 10.3389/fpubh.2026.1789136

**Published:** 2026-03-27

**Authors:** Yan-hua Li, Ping-shan Jiang, Lu-xi Bai, Chun-jiao Xie, Yuan Zhao, Qing Li, Wen-feng Zeng, Feng Zhu

**Affiliations:** 1 Occupational Health Surveillance Centre, The Affiliated Guangzhou Twelfth People's Hospital, Guangzhou Medical University, Guangzhou, China; 2 Occupational Health Management Section, The Affiliated Guangzhou Twelfth People's Hospital, Guangzhou Medical University, Guangzhou, China; 3 Central Laboratory, The Affiliated Guangzhou Twelfth People's Hospital, Guangzhou Medical University, Guangzhou, China

**Keywords:** Fas, gene polymorphism, hearing loss, noise, single nucleotide polymorphisms (SNPs)

## Abstract

**Introduction:**

The FAS gene, as a core regulator of the cell apoptosis pathway, has attracted considerable attention for its potential role in maintaining the homeostasis of inner ear hair cells and resisting noise-induced oxidative damage. This study aims to explore and verify the relationship between FAS gene polymorphisms and NIHL susceptibility.

**Subjects and methods:**

In this case-control study, a total of 364 noise-exposed workers were enrolled, comprising 156 NIHL cases and 208 normal-hearing controls.Genotyping of SNP loci in the FAS gene was performed using the MassArray system. Multivariate logistic regression analysis was used to evaluate the association between the identified SNPs and the susceptibility to noise-induced hearing loss (NIHL).

**Results:**

The genotype distributions in both groups were in Hardy-Weinberg equilibrium. Multivariate logistic regression analysis after adjustment for confounders showed that the T allele at the rs1468063 locus was associated with a significantly increased risk of NIHL compared to the C allele (OR = 1.79, 95% CI = 1.32–2.44, *p* < 0.001). In the codominant model, individuals with the CT (OR=2.14) and TT (OR = 3.86) genotypes had a higher risk compared to those with the CC genotype. Consistent with this, the recessive model (TT vs. CC + CT) also indicated that the TT genotype conferred a higher risk (OR = 2.27); for rs1800682 locus, the increased risks of NIHL were associated with G allele compared with A allele, the risk of NIHL was 2.81-fold higher in individuals with the GG genotype compared with those with the AA genotype.

**Conclusion:**

T allele at rs1468063 and G allele at rs1800682 of FAS gene were independently associated with the increased risk for NIHL, and these alleles may be potential biomarkers for early identification and risk stratification to NIHL.

## Introduction

Noise-induced hearing loss (NIHL) is a common sensor neural hearing impairment, second only to age-related hearing loss in terms of prevalence (1). Current research consistently indicates that NIHL is a complex condition resulting from the interplay of multiple factors, including age, sex, noise exposure intensity, duration of exposure, smoking, and alcohol consumption, with long-term exposure to high-intensity noise being the most significant contributor to NIHL (2). As early as 1995, researchers observed that among individuals exposed to the same noise environment for the same duration, only a subset developed hearing loss, and the severity of hearing loss varied (3). Further studies have confirmed significant inter-individual differences in susceptibility to NIHL under identical noise exposure conditions (4, 5). This suggests that genetic factors may play a crucial role in the development and progression of NIHL. The underlying mechanisms may involve the regulation of the inner ear’s sensitivity to oxidative stress, immune-inflammatory responses, ion homeostasis, and apoptosis, thereby influencing the occurrence and development of NIHL.

In recent years, studies based on molecular genetics have identified several candidate genes associated with susceptibility to noise-induced hearing loss (NIHL) (6), such as catalase (CAT) (7), histone deacetylase 2 (8), cadherin 23 (CDH23) (9), and apoptosis-related genes (10, 11). Among these, the FAS gene, as a core regulator of the cell apoptosis pathway, has attracted considerable attention for its potential role in maintaining the homeostasis of inner ear hair cells and resisting noise-induced oxidative damage (12). However, given that the FAS gene belongs to the tumor necrosis factor receptor (TNFR) superfamily and plays a key role in the physiological regulation of programmed cell death, the research focus of most scholars has been on malignant tumors and neurodegenerative diseases, with relevant studies further confirming this perspective. That is, polymorphisms in the FAS gene can influence the transcriptional activity of the gene or the expression levels of the protein, thereby altering the threshold for apoptosis and being closely related to the susceptibility of various diseases, such as tumors and neurodegenerative diseases (13–15). Currently, there are few studies on the relationship between FAS gene polymorphisms and NIHL susceptibility. This study, designed as a case–control study, aims to explore and verify the relationship between FAS gene polymorphisms and NIHL susceptibility. It is expected to provide a theoretical basis for the early screening of high-risk and susceptible populations for NIHL, individualized protection, as well as the prevention, treatment, and drug development for NIHL. Meanwhile, it offers a new perspective for genetic research on noise-induced hearing loss.

## Subjects and methods

### Selection of participants

Participants were selected from workers exposed to occupational noise who underwent occupational health examinations at our hospital between June and December 2024. All participants were recruited from multiple enterprises, covering diverse industrial sectors, including: 124 automobile manufacturing workers (34.1%), 93 shipyard workers (25.5%), 62 aircraft maintenance workers (17.0%), 49 road maintenance workers (13.5%), and 36 glass manufacturing workers (9.9%). Inclusion criteria were: (1) work tenure in noise-exp Inclusion criteria: (1) Work tenure in noise-exposed occupations ≥1 year; (2) 8-h equivalent noise level at the workplace ≥80 dB (A); (3) Informed consent from the study subjects. Exclusion criteria: (1) Presence of conductive or mixed hearing loss as determined by pure-tone audiometry;

(2) History of otitis media, head trauma, hereditary deafness, blast-induced hearing loss, or use of ototoxic medications. A case–control study design was employed. Based on the inclusion and exclusion criteria, and considering the completeness of hearing test results, workplace noise measurement data, questionnaire survey results, and single nucleotide polymorphism (SNP) genotyping results, 156 subjects were ultimately assigned to the case group. Additionally, 208 workers with normal hearing and similar age, noise exposure duration, and noise exposure levels to the case group were selected as the control group. This study was approved by the Medical Ethics Committee of our hospital (Approval No. 2023067), all participants provided informed consent, and all methods in this study were performed in accordance with the Declaration of Helsinki.

### Questionnaire survey

A questionnaire specifically designed for workers exposed to occupational noise was developed according to the study requirements. The main content included general demographic information, past medical history, family history, medication use history, lifestyle habits (in this study, smoking was defined as consuming at least one cigarette per day for ≥6 months; alcohol consumption was defined as drinking at least once per week for over 1 year), occupational history, and exposure history to occupational hazards. The questionnaires were administered and supervised by members of the research team who had undergone unified training.

### Pure-tone audiometry

All study subjects underwent pure-tone audiometry 48 h after being removed from noise exposure. In accordance with the “Diagnostic Criteria for Occupational Noise-Induced Hearing Loss” (GBZ 49–2014), the tests were conducted by professional physicians in a soundproof room with a background noise level of < 25 dB (A), using the Madsen Itera audiometer from Madsen, Denmark. The pure-tone air conduction hearing thresholds were measured for both ears at frequencies of 0.5, 1.0, 2.0, 3.0, 4.0, and 6.0 kHz. The hearing thresholds were adjusted for age and sex according to Appendix A of GBZ 49–2014. After excluding other diseases or factors, subjects with a corrected hearing threshold shift > 25 dB in any of the 3, 4, or 6 kHz frequencies in either ear were diagnosed with NIHL and assigned to the case group.

### On-site noise measurement

The noise exposure levels of the study subjects were measured in accordance with the “Measurement of Physical Factors in the Workplace - Part 8: Noise” (GBZ/T 189.8–2007). The EDGE personal noise dosimeter from the UK-based company CASELLA was used to measure and calculate the noise exposure levels of the study subjects, specifically the 8-h equivalent noise level (LEX, 8 h).

### DNA extraction

Five milliliters of fasting venous blood were collected from the study subjects in the morning and anticoagulated with heparin. DNA was extracted using the Blood Genomic DNA Extraction Kit from Nanjing Zhongke Baili Company. The quality of all DNA samples was assessed using the NanoDrop2000 spectrophotometer for OD value measurement and 1.25% agarose gel electrophoresis to ensure that the DNA met the requirements for SNP genotyping.

### SNP genotyping of the target gene

Potential functional SNP sites of the FAS gene were selected through the dbSNP database^1^ and the UCSC database.^2^ The selection criteria were as follows: (1) a minor allele frequency of ≥0.1 in the Han Chinese population; (2) the sites located in functional regions such as the promoter, coding region, splice sites at the junction of introns and exons; (3) linkage disequilibrium value r2 > 0.8; (4) SNP sites reported in domestic and international literature to be associated with FAS and NIHL. The MassArray SNP genotyping technology from Agena Bioscience (United States) was used to detect FAS gene polymorphisms. The primer information used in the experiment is shown in Table 1.

**Table 1:** Primer sequences for SNP amplification and extension.

SNP loci	Primer sequence(5′-3′)	Length(bp)
rs1468063		
1st-PCRP	ACGTTGGATGCGTAGGATAGTAGTAAGGAG	96
2nd-PCRP	ACGTTGGATGCATCTGGATTTAGGAATTGC	
UEP_SEQ	CCCCTGAATTGCTCTTGTCATACCC	
rs2862833		
1st-PCRP	ACGTTGGATGTACCTTCTAAGGGATCCAAG	108
2nd-PCRP	ACGTTGGATGGGGCTTGCTTTTTGGTTTTG	
UEP_SEQ	TGATGCTAAATATAACTTGTCTTT	
rs1800682		
1st-PCRP	ACGTTGGATGTCCCTTTTCAGAGCCCTATG	118
2nd-PCRP	ACGTTGGATGTTGTGGCTGCAACATGAGAG	
UEP_SEQ	AGGCTCACAGACGTT	

### Statistical analysis

Statistical analyses were performed using SPSS 20.0 software. After normality testing, continuous variables that followed a normal distribution were expressed as means ± standard deviations (SD). Comparisons between two groups were conducted using the t-test. For continuous variables that did not follow a normal distribution, the median and interquartile range (M [P25, P75]) were used, and comparisons between groups were performed using the Wilcoxon rank-sum test. Categorical variables were expressed as percentages and proportions, and comparisons between groups were conducted using the chi-square (χ^2^) test. The Hardy–Weinberg equilibrium was assessed using the goodness-of-fit test. Binary unconditional logistic regression analysis was used to calculate the odds ratios (OR) and 95% confidence intervals (CI). A *p*-value less than 0.05 divided by the number of comparisons was considered statistically significant. Based on the minor allele frequencies in the control group (rs1468063 T = 0.399, rs1800682 G = 0.411), the observed odds ratios (rs1468063 OR = 1.79, rs1800682 OR = 1.61), with a two-sided significance level of *α* = 0.05 and sample sizes of 208 controls and 156 cases, the estimated statistical power was 85.3% for the rs1468063 T allele and 79.8% for the rs1800682 G allele. The slightly lower power for the rs1800682 locus (79.8%) is still acceptable and close to the conventional 80% threshold. These calculations indicate that the study was adequately powered to detect the main effects reported.

## Results

### Basic characteristics of study subjects

All study subjects were male Han Chinese workers, totalling 364 individuals. The average age was 35.57 ± 8.65 years, and the average duration of exposure to occupational hazards was 9.41 ± 7.18 years. In the case group, the average age was 35.28 ± 8.89 years, and the average duration of exposure was 10.00 ± 7.07 years. In the control group, the average age was 32.28 ± 8.25 years, and the average duration of exposure was 8.96 ± 7.25 years. Comparisons between the two groups in terms of noise exposure duration, smoking status, alcohol consumption, physical exercise, and noise exposure intensity (LEX,8 h) revealed no statistically significant differences (*p* > 0.05), indicating that the basic characteristics of the two groups were comparable. Detailed information is presented in Table 2.

**Table 2:** Baseline characteristics of case and control groups.

Variables	Control(*n* = 208)	Case(*n* = 156)	*c*^2^/*Z*	*p*
Age(year)	32.28 ± 8.25	35.28 ± 8.89	−3.31	0.001
Years of service as a victim(year)	8.96 ± 7.25	10.00 ± 7.07	−1.38	0.170
Smoking			2.642	0.0450
Never smoked	94(45.2)	58(37.2)		
Occasional cigarette	24(11.5)	23(14.7)		
Regular smoking	68(32.7)	55(35.3)		
Have given up smoking	22(10.6)	20(12.8)		
Alcohol consumption			0.907	0.824
Never drink alcohol.	99(47.6)	72(46.2)		
Occasional drinking	99(47.6)	75(48.1)		
Regular consumption of alcohol	4(1.9)	2(1.3)		
Sober	6(2.9)	7(4.5)		
Exercise habit			0.512	0.774
Never worked out	19(9.1)	11(7.1)		
An occasional workout	150(72.1)	115(73.7)		
Regular exercise	39(18.8)	30(19.2)		
Noise intensity(dB)	82.53 ± 2.77	82.27 ± 2.31	0.966	0.335

### Hardy–Weinberg equilibrium test

The genotype frequency distribution of the SNP loci in both groups was analyzed using the chi-square goodness-of-fit test, revealing that the observed genotype frequencies were in accordance with the Hardy–Weinberg equilibrium (*p* > 0.05). This indicates that the SNP genotypes were in a state of equilibrium, and the subjects selected for this study were representative (Table 3).

**Table 3:** Hardy–Weinberg equilibrium testing for SNP genotypes.

Group(n)	Value	rs1468063					rs2862833					rs1800682				
		CC	CT	TT	χ*^2^*	*p*	GG	GA	AA	χ*^2^*	*p*	AA	AG	GG	χ*^2^*	*p*
Control(208)	Observed	69	112	27	1.53	0.466	64	112	32	1.12	0.572	67	111	30	1.04	0.596
	Expected	75	100	33			69	101	37			72	101	35		
Case(156)	Observed	26	89	41	1.90	0.387	38	76	42	0.05	0.975	35	75	46	0.05	0.974
	Expected	32	77	47			37	78	41			34	77	45		

### Genotype and allele frequency distribution of SNP loci between the two groups

The comparison of genotype and allele frequencies at the rs1468063 locus revealed statistically significant differences between the case and control groups for the CC, CT, and TT genotypes, as well as for the C and T alleles (*p* < 0.01). For the rs2862833 locus, a statistically significant differences was observed in the allele frequencies between the two groups (*p* = 0.016), although the genotype frequency distribution did not reach the pre-defined significance threshold after Bonferroni correction (*p* = 0.022). Similarly, the comparison of genotype and allele frequencies at the rs1800682 locus showed statistically significant differences for the AA, AG, and GG genotypes, as well as the A and G alleles (*p* < 0.01). Detailed information is presented in Table 4.

**Table 4:** Comparison of genotypes and allele frequencies of SNP loci between the two groups.

SNP loci	Group(n)	Allele	Genotype [n (%)]					Allele [n (%)]			
			PP	PQ	QQ	χ^2^	*p*	P	Q	χ^2^	*p*
rs1468063	Control(208)	T/C	69(33.17)	112(53.85)	27(12.98)	17.914	<0.01	250(60.10)	166(39.90)	15.928	<0.01
	Case(156)		26(16.67)	89(57.05)	41(26.28)			141(45.19)	171(54.81)		
rs2862833	Control(208)	A/G	64(30.77)	112(53.85)	32(15.38)	7.599	0.022	240(57.69)	176(42.31)	5.778	0.016
	Case(156)		38(24.36)	76(48.72)	42(26.92)			152(48.72)	160(51.28)		
rs1800682	Control(208)	G/A	67(32.21)	111(53.37)	30(14.42)	13.217	<0.01	245(58.89)	171(41.11)	11.057	<0.01
	Case(156)		35(22.44)	75(48.08)	46(29.49)			145(46.47)	167(53.53)		

1. For rs1468063, P=CC, Q = T; For rs2862833, P = G, Q = A; For rs1800682, P = A, Q = G. 2. Bonferroni correction for three independent SNP loci was applied, setting the significance threshold at α = 0.017. *p*-values below this threshold are considered statistically significant after correction.

### Association between FAS gene polymorphisms and susceptibility to NIHL

Individuals with the T allele at the rs1468063 locus had a 1.79-fold increased risk of developing NIHL compared with those carrying the C allele (OR = 1.79, 95% CI = 1.32–2.44, *p* < 0.001), suggesting that the T allele may be a risk factor for NIHL. Compared with wild-type homozygotes (CC), heterozygotes (CT) had a 2.14-fold increased risk (OR = 2.14, 95% CI: 1.25–3.68, *p* = 0.006), while mutant homozygotes (TT) had a 3.86-fold increased risk (OR = 3.86, 95% CI: 1.96–7.62, *p* < 0.001). Individuals with the TT homozygous genotype had a 2.27-fold increased risk of NIHL compared to those carrying at least one C allele (CC + CT; OR = 2.27, 95% CI: 1.30–3.94, *p* = 0.004). Individuals carrying at least one T allele (CT + TT) had a 2.48-fold increased risk of NIHL compared to those with the CC genotype (OR = 2.48, 95% CI: 1.47–4.18, *p* < 0.001). The T allele at the rs1468063 locus is an independent risk factor for NIHL, and the risk is more pronounced in the homozygous state. Individuals carrying the A allele at the rs2862833 locus had a 1.39-fold increased risk of NIHL compared to those carrying the G allele (OR = 1.39, 95% CI: 1.03–1.88, *p* = 0.032), but the *p* value exceeded the corrected threshold (*α* = 0.017). In addition, no statistically significant association were observed in the codominant model,recessive model and dominant model. For the rs1800682 locus, individuals carrying the G allele had a 1.61-fold increased risk of NIHL compared with those carrying the A allele (OR = 1.61, 95% CI = 1.19–2.18, *p* = 0.002), suggesting that the G allele may be a risk factor for NIHL. The risk of NIHL was 2.81-fold higher in individuals with the GG genotype compared with those with the AA genotype (OR = 2.81, 95% CI = 1.50–5.28, *p <* 0.001). Whereas the AG genotype showed no significant association (adjusted OR = 1.22, 95% CI: 0.73–2.05, *p* = 0.448). The GG homozygous genotype conferred a 2.47-fold increased risk compared to the combined AA+AG genotypes (adjusted OR = 2.47, 95% CI: 1.45–4.18, *p* < 0.001). No significant association was observed for the AG + GG combined genotype compared to the AA genotype (*p* = 0.075; Table 5).

**Table 5:** Logistic regression analysis of gene polymorphism and susceptibility to NIHL.

Genetic models	Genotypes	Initial OR(95%CI)	P(Initial)	Adjusted OR(95%CI)*	P(Adjusted)*
rs1468063					
Codominant	CT/CC	2.11(1.24–3.58)	0.006	2.14(1.25–3.68)	0.006
	TT/CC	4.03(2.08–7.82)	<0.001	3.86(1.96–7.62)	<0.001
Recessive	TT/CC + CT	2.39(1.39–4.10)	0.002	2.27(1.30–3.94)	0.004
Dominant	CT + TT/CC	2.47(1.48–4.19)	<0.001	2.48(1.47–4.18)	<0.001
Alleles	T/C	1.83(1.36–2.46)	<0.001	1.79(1.32–2.42)	<0.001
rs2862833					
Codominant	GA/GG	1.10(0.67–1.80)	0.708	1.11(0.67–1.84)	0.685
	AA/GG	2.15(1.17–3.96)	0.013	2.02(1.08–3.77)	0.027
Recessive	AA/GG + GA	2.03(1.21–3.40)	0.007	1.89(1.11–3.21)	0.019
Dominant	GA + AA/GG	1.33(0.82–2.13)	0.227	1.32(0.82–2.12)	0.260
Alleles	A/G	1.44(1.07–1.93)	0.016	1.39(1.03–1.88)	0.032
rS1800682					
Codominant	AG/AA	1.29(0.78–2.14)	0.316	1.22(0.73–2.05)	0.448
	GG/AA	2.94(1.59–5.43)	<0.001	2.81(1.50–5.28)	<0.001
Recessive	GG/AA+AG	2.48(1.48–4.16)	<0.001	2.47(1.45–4.18)	<0.001
Dominant	AG + GG/AA	1.63(0.98–2.57)	0.041	1.56(0.96–2.54)	0.075
Alleles	G/A	1.65(1.23–2.22)	<0.001	1.61(1.19–2.18)	0.002

*Adjusted for age, smoking status, alcohol consumption, exercise habit, and noise exposure intensity in the multivariate logistic regression models.

## Discussion

In this study, we demonstrate that polymorphisms in the Fas gene modulate susceptibility to NIHL. Specifically, the T allele at rs1468063 and the G allele at rs1800682 were independently associated with the increased risk of NIHL. These findings not only substantiate ongoing epidemiologic inquiries into NIHL but furnish novel mechanistic leads and preventive targets for precision interventions against this debilitating condition.

The susceptibility to NIHL refers to the genetic predisposition of an individual to develop NIHL upon exposure to noise, which plays a significant role in the occurrence and progression of NIHL (16). The pathogenesis of NIHL has been extensively studied. Most scholars believe that following noise exposure; individuals can trigger a series of complex molecular cascades within inner ear hair cells through pathways such as oxidative stress induction, inflammatory factor release, and mitochondrial dysfunction, thereby activating apoptosis signaling pathways and ultimately leading to irreversible damage and loss of hair cells (17–19). Moreover, NIHL is a polygenic disease involving multiple SNPs in several genes. Molecular genetic studies have revealed that the polymorphisms of various apoptosis-related genes are closely associated with the susceptibility to NIHL (6, 20–22). In the apoptosis mechanism of hair cells, the FAS gene, as a key regulator of apoptosis, encodes the FAS receptor, which belongs to the tumor necrosis factor receptor (TNFR) superfamily and plays a central role in the regulation of programmed cell death. Upon binding of the FAS receptor to its ligand FasL, the death domain (DD) of FAS interacts with the Fas-associated death domain protein (FADD), recruiting Caspase-8 to form the death-inducing signaling complex (23, 24). This complex further activates downstream apoptotic execution enzymes such as Caspase-3, ultimately leading to hair cell apoptosis. SNPs in the FAS gene may affect its function and thereby alter the sensitivity of hair cells to noise stimulation. However, current research on the association between FAS gene polymorphisms and NIHL susceptibility remains limited. Therefore, this study selected three key loci of the FAS gene (rs1468063, rs2862833, and rs1800682) to verify their potential association with NIHL susceptibility.

The rs1468063 locus, located in the 3′ untranslated region (3’UTR) of the FAS gene, is SNP associated with various diseases, including Alzheimer’s disease (AD), noise-induced hearing loss (NIHL), and gastric cancer (13–15). These findings suggest that genetic variation at the rs1468063 locus may play a significant role in the pathogenesis of multiple diseases. In this study, we observed that the T allele at rs1468063 was associated with a significantly increased risk of NIHL. Specifically, compared to the CC genotype, individuals with the CT and TT genotypes exhibited a 2.14-fold and 3.86-fold higher risk, respectively. Our findings are consistent with those of Wu et al. (25), who also reported the TT variant genotypes were related to NIHL susceptibility. However, this conclusion contrasts with the previous findings (26), who reported that the TT genotype at the rs1468063 locus is associated with a reduced risk of NIHL. The rs2862833 locus, located in the non-coding region of the FAS gene, exhibits a SNP characterized by an A/G variation, resulting in three genotypes: AA, AG, and GG. In two previous studies investigating the association between the rs2862833 genotype and susceptibility to NIHL (25, 26), which suggested that the AA genotype at the rs2862833 locus may reduce the risk of NIHL by inhibiting the over activation of the FAS signaling pathway, thereby decreasing noise-induced cochlear cell apoptosis. This genotype has been proposed as a potential protective marker for NIHL in the Chinese population, particularly in the screening of individuals exposed to high levels of occupational noise. However, our study found no significant association between the polymorphism at the rs2862833 locus and the risk of NIHL, indicating that this polymorphism may not be related to the genetic susceptibility to NIHL in our cohort. Beyond differences in sample size or population selection, several unverified factors may contribute to the observed inconsistencies between our findings and those of previous studies (particularly regarding the rs1468063 and rs2862833 loci). First, gene–environment and gene–gene interactions are likely critical modulators of genetic risk for NIHL, a complex polygenic disease. Our study focused on the main effects of individual loci and did not evaluate such higher-order interactions. Therefore, variations in the distribution of these interactive factors, such as the age structure of the cohort, the rate of personal protective equipment use, or the frequency of interacting genotypes at other loci between our population and other studies—may explain the divergent conclusions we observed. Second, heterogeneity in noise exposure assessment and NIHL phenotype definition may also play a role. Although all studies selected workers with occupational noise exposure, there are subtle but important differences in exposure metrics (e.g., using a simple 8-h average vs. cumulative noise exposure) and the precise thresholds used to define hearing loss cases. Such variations can affect the statistical power to detect genetic associations and may contribute to the observed variability in results. Third, the possibility of differences in underlying linkage disequilibrium (LD) patterns cannot be entirely excluded. The rs1468063 and rs2862833 SNPs may not be the causal variants themselves, but rather genetic markers in LD with the true functional variants elsewhere in the FAS gene or its regulatory regions. If the LD structure between these markers and the causal variants differs subtly between study cohorts—even within the same Han Chinese population—it could lead to inconsistent association signals. This underscores the need for future fine-mapping or functional studies to identify the precise causal variants and elucidate their biological effects.

Turning to the rs1800682 locus, it is a single nucleotide polymorphism in the promoter region of the Fas gene, located at position −670 and involving an A/G variation. This SNP has been associated with the risk of various diseases, including breast cancer, colorectal cancer, systemic lupus erythematosus, cervical cancer, and hepatocellular carcinoma. Currently, most studies have focused on the rs1468063 and rs2862833 loci of the Fas gene, while research on the association between the rs1800682 locus and the risk of NIHL has not been reported. In this study, we found that individuals carrying the G allele had a 1.61-fold increased risk of NIHL compared with those carrying the A allele. Additionally, individuals with the GG genotype at the rs1800682 locus had a 2.81-fold increased risk of NIHL compared with those with the AA genotype, while those with the GG genotypes had a 2.47-fold increased risk compared with those with the AA + AG genotype. Our findings suggested that the G allele may be a risk factor for NIHL. Mechanistically, the A/G variation at rs1800682 may influence the transcriptional activity of the FAS gene by altering the transcriptional activation mediated by the SP1/STAT1 complex, thereby affecting the process of cell apoptosis and consequently influencing disease occurrence and progression (27). This study aimed to explore the relationship between polymorphisms in the FAS gene and the risk of NIHL. To our knowledge, this is the first study to report an association between the rs1800682 G allele and an increased risk of NIHL, these findings should be considered exploratory and require validation in independent cohorts.

**Figure 1:**
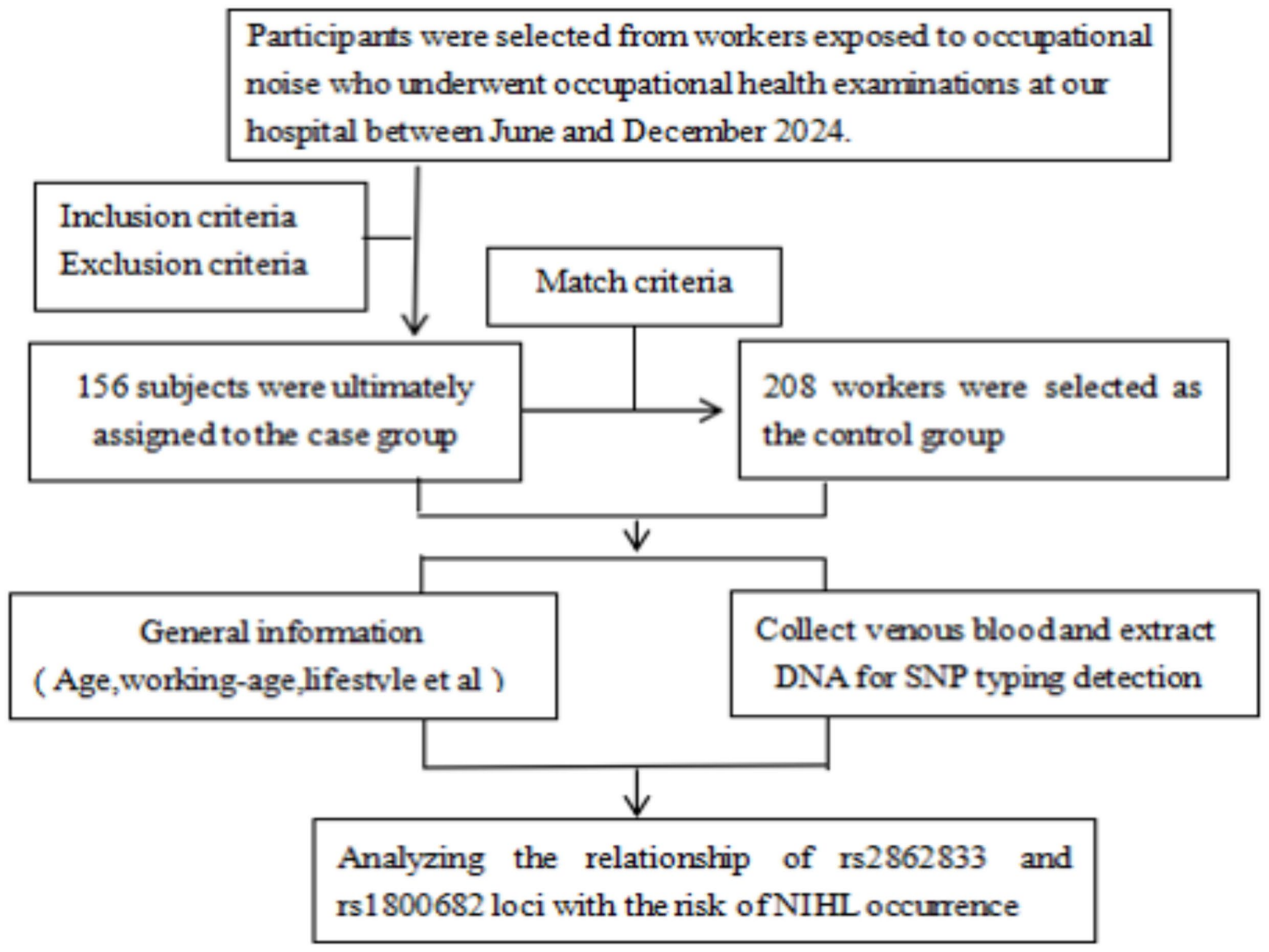
Flow diagram of participants selected for analyses in this study.

It is noteworthy that baseline differences were observed between the case and control groups regarding age (35.28 ± 8.89 years vs. 32.28 ± 8.25 years, *p* = 0.001) and smoking status (*p* = 0.045; Table 2). Age is a well-established risk factor for hearing loss, including NIHL, as age-related cochlear degeneration may enhance susceptibility to noise exposure (28). Similarly, smoking has been implicated in NIHL pathogenesis through its vasoconstrictive effects on cochlear blood flow and its role in promoting oxidative stress (29). The presence of these imbalances could potentially confound the observed associations between FAS gene polymorphisms and NIHL risk. To address this concern, we employed multivariate logistic regression analysis adjusting for age, smoking, and other potential confounders (Table 5). The independent associations of the rs1468063 T allele and rs1800682 G allele with increased NIHL risk persisted after these adjustments, suggesting that the observed genetic effects are not merely artifacts of confounding by age or smoking (Figure 1).

Despite these intriguing findings, several limitations of this study should be acknowledged. First, the relatively modest sample size (*n* = 364) may limit the statistical power to detect weak associations. Second, the study population was restricted to Chinese Han male workers. While this minimizes population stratification, it limits the generalizability of our findings to other ethnicities or females. Nevertheless, our study has several notable strengths. To our knowledge, this is the first study to report an association between the rs1800682 G allele and an increased risk of NIHL. Although these findings are preliminary and require validation in independent cohorts, they may offer novel insights into the genetic basis of NIHL. Future research should focus on larger, multi-center, population-based studies to comprehensively elucidate the role of FAS gene polymorphisms in NIHL susceptibility.

## Conclusion

In conclusion, this study provides evidence that polymorphisms in the FAS gene are associated with susceptibility to NIHL in a Chinese occupational population. The T allele at rs1468063 and the G allele at rs1800682 were identified as independent risk factors for NIHL. Our findings particularly reported an association between rs1800682 and NIHL, offer new insights into the genetic basis of NIHL and suggest potential biomarkers for early risk stratification.

## Data Availability

The original contributions presented in the study are included in the article, further inquiries can be directed to the corresponding author.

## Footnotes

1
http://www.ncbi.nlm.nih.gov/snp/

2
http://genome.ucsc.edu/
